# Clinical implications of *Plasmodium* resistance to atovaquone/proguanil: a systematic review and meta-analysis

**DOI:** 10.1093/jac/dkx431

**Published:** 2017-12-11

**Authors:** Henry M Staines, Rebekah Burrow, Beatrix Huei-Yi Teo, Irina Chis Ster, Peter G Kremsner, Sanjeev Krishna

**Affiliations:** 1 Centre for Diagnostics and Antimicrobial Resistance, Institute for Infection & Immunity, St George’s University of London, London, UK; 2 Institute for Infection & Immunity, St George's University of London, London, UK; 3 Institut für Tropenmedizin Universitätsklinikum Tübingen, Tübingen, Germany; 4 Centre de Recherches Médicales de Lambaréné, Lambaréné, Gabon; 5 St George’s University Hospitals NHS Foundation Trust, London, UK

## Abstract

**Background:**

Atovaquone/proguanil, registered as Malarone^®^, is a fixed-dose combination recommended for first-line treatment of uncomplicated *Plasmodium falciparum* malaria in non-endemic countries and its prevention in travellers. Mutations in the cytochrome *bc_1_* complex are causally associated with atovaquone resistance.

**Methods:**

This systematic review assesses the clinical efficacy of atovaquone/proguanil treatment of uncomplicated malaria and examines the extent to which codon 268 mutation in cytochrome *b* influences treatment failure and recrudescence based on published information.

**Results:**

Data suggest that atovaquone/proguanil treatment efficacy is 89%–98% for *P. falciparum* malaria (from 27 studies including between 18 and 253 patients in each case) and 20%–26% for *Plasmodium vivax* malaria (from 1 study including 25 patients). The *in vitro P. falciparum* phenotype of atovaquone resistance is an IC_50_ value >28 nM. Case report analyses predict that recrudescence in a patient presenting with parasites carrying cytochrome *b* codon 268 mutation will occur on average at day 29 (95% CI: 22, 35), 19 (95% CI: 7, 30) days longer than if the mutation is absent.

**Conclusions:**

Evidence suggests atovaquone/proguanil treatment for *P. falciparum* malaria is effective. Late treatment failure is likely to be associated with a codon 268 mutation in cytochrome *b*, though recent evidence from animal models suggests these mutations may not spread within the population. However, early treatment failure is likely to arise through alternative mechanisms, requiring further investigation.

## Introduction

Infection with *Plasmodium* spp. is a major cause of mortality worldwide, causing 235 000–639 000 deaths in 2015 and 148 000 000–304 000 000 clinical cases of malaria. Most cases are in endemic countries, although malaria is also one of the most frequent causes of morbidity in travellers returning to non-endemic countries. Atovaquone/proguanil (Malarone^®^) is a fixed-dose combination often used as a first-line treatment for uncomplicated *Plasmodium falciparum* infections in non-endemic countries.^1^^,^^2^ It has been used on a large scale as a treatment in areas where treatment failures of artemisinin combination therapies (TFACT)^3^ are problematic.^4^ It is now considered a first-line prophylaxis against malaria for travellers^5^ and particularly military personnel whose experience of adverse events with mefloquine prophylaxis is becoming increasingly recognized.^6^ Atovaquone/proguanil is also being studied in a new chemo-vaccination strategy where individuals are exposed to *P. falciparum* sporozoites and then take atovaquone/proguanil to treat pre-symptomatic infections and generate antimalarial immunity (P. G. Kremsner, unpublished). Taken together with the recent expiry of patent protection for Malarone^®^, usage of atovaquone/proguanil is likely to rise in the future.

**Figure 1.**
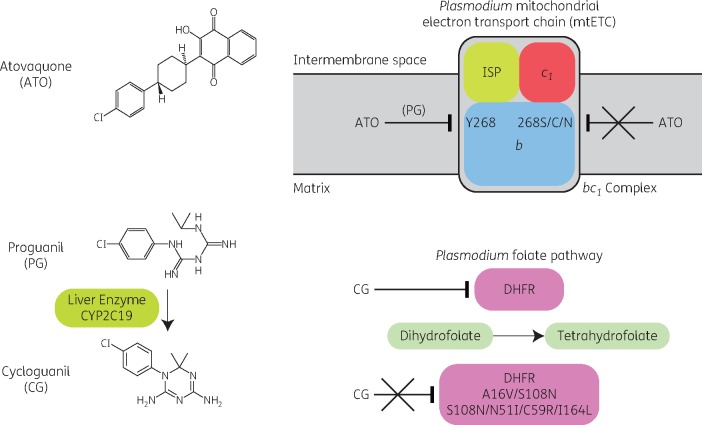
Mechanisms of action and resistance to atovaquone/proguanil. Structures of atovaquone, proguanil and cycloguanil are shown. Atovaquone targets cytochrome *b* in the *bc_1_* complex [formed by cytochromes *b* and *c_1_* and the Rieske iron–sulphur protein (ISP)] of the *Plasmodium* mitochondrial electron transport chain. The mitochondrial electron transport chain is located on the inner membrane of mitochondria, separating the intermembrane space (the space between the outer and inner membranes) from the centrally located matrix. Atovaquone works in synergy with proguanil, but its activity is reduced by mutations in cytochrome *b* (and in particular Y268S/C/N). Proguanil is metabolized to cycloguanil by the liver enzyme CYP2C19. Cycloguanil targets the enzyme DHFR in the *Plasmodium* folate pathway. Activity of cycloguanil is reduced by mutations in DHFR, including A16V/S108N and S108N/N51I/C59R/I164L. This figure appears in colour in the online version of *JAC* and in black and white in the print version of *JAC*.

Atovaquone is a hydroxynaphthoquinone that selectively inhibits the mitochondrial electron transport chain at the cytochrome *bc_1_* complex of malaria parasites (Figure 1).^7^ This mechanism of antiparasitic activity is complemented by the individual actions of proguanil and its metabolite, cycloguanil (Figure 1). Proguanil itself has no direct effects on the parasite, but it enhances atovaquone’s ability to collapse the membrane potential of malaria parasites by sensitizing mitochondria to atovaquone.^8^ Proguanil is converted into cycloguanil by the hepatic CYP2C19 system and cycloguanil inhibits parasite dihydrofolate reductase (DHFR), which is essential for folate production and parasite replication.^9^

Several mechanisms can potentially influence the efficacy of atovaquone/proguanil for treatment. Mutations in *P. falciparum* cytochrome *b* (*PfCYTb*) (in particular leading to Y268S/C/N) cause atovaquone resistance both *in vitro* and *in vivo*.^10–12^ Interestingly, a recent report, using a rodent model of malaria infection, describes that mutations in *Plasmodium berghei CYTb* are lethal during transmission of the parasite in the mosquito vector.^13^ This suggests that these mutations may not be able to spread within a population, although this hypothesis has yet to be demonstrated for *P. falciparum* in the field. Cycloguanil resistance in parasites is conferred by multiple mutations in *DHFR*. Polymorphisms in host *CYP2C19* also affect proguanil metabolism and can lower cycloguanil concentrations.^14^

Reports of frequencies of treatment failure associated with atovaquone/proguanil vary, although the risk of failure has not been systematically examined particularly with respect to mutations at codon 268 of *PfCYTb*. In this systematic review, we examine all original *in vivo* data where atovaquone/proguanil was used exclusively to treat malaria and relate findings on risk of recrudescence to mutations in *PfCYTb* and available results from *in vitro* assays. We also estimate clinical efficacy of atovaquone/proguanil treatment of uncomplicated malaria. Results may impact on existing guidelines for the treatment of uncomplicated malaria.

## Methods

### Search strategy and selection criteria

This systematic review was registered at PROSPERO (number CRD42015020757) on 25 February 2015 and updated on 13 October 2017.

PubMed (1966–present) and ScienceDirect (1823–present) were interrogated on the 19 May 2015 with the following search strategy {[(Atovaquone AND Proguanil) OR (Malarone)] AND (falciparum OR vivax OR ovale OR malariae OR knowlesi)}. Records were assessed for eligibility using title, or title and abstract. Eligible records were screened for duplicates and full-text obtained for the remaining records that were then reassessed for eligibility. Data were extracted from these articles by two reviewers and tabulated. Inclusion and exclusion criteria and extracted data variables are summarized in the Supplementary Methods (available as Supplementary data at *JAC* Online).

### Group studies

Two reviewers assessed group study eligibility and the risk of bias in the trials using the modified Cochrane risk of bias tool.^15^ Six domains of bias were assessed with regard to selection, performance, detection, attrition, reporting and other, and the risk of bias deemed as low, medium, high or unclear. The information was not used to exclude studies from this review, but the assessment fed into the interpretation of results.

For all group studies, the total numbers of patients enrolled into each treatment arm, those followed up to 28 days and those with treatment failure or recrudescence were extracted and combined to obtain the proportion of patients for whom treatment had been successful in the ITT and PP populations. For randomized controlled trials (RCT), this information was also extracted for the comparator antimalarial arm(s) to allow meta-analyses (pooled ORs of the alternative intervention versus atovaquone/proguanil).

A random effects model to derive a pooled OR of treatment success for atovaquone/proguanil versus comparator treatments, if appropriate, was applied and interpreted in conjunction with a corresponding heterogeneity χ^2^ test and additional sensitivity analyses undertaken (Supplementary Methods). Data were analysed with Stata version 14, with forest plots generated in Review Manager version 5.3.

### In vitro/ex vivo studies

For *in vitro*/*ex vivo* studies, no mathematical synthesis was carried out.

### Case reports

Preliminary exploratory analyses examined all the variables using graphs and statistical tests for comparisons according to the nature of the data. Regression techniques were implemented to understand potential associations between pretreatment parasitaemia and (i) minimum days to recrudescence (defined as the length of time in days since treatment to the occurrence of clinical signs or parasitological diagnosis, whichever came first), and (ii) parasitaemia at recrudescence with presence of mutation in *PfCYTb* codon 268 in both cases (Supplementary Methods).

## Results

**Figure 2.**
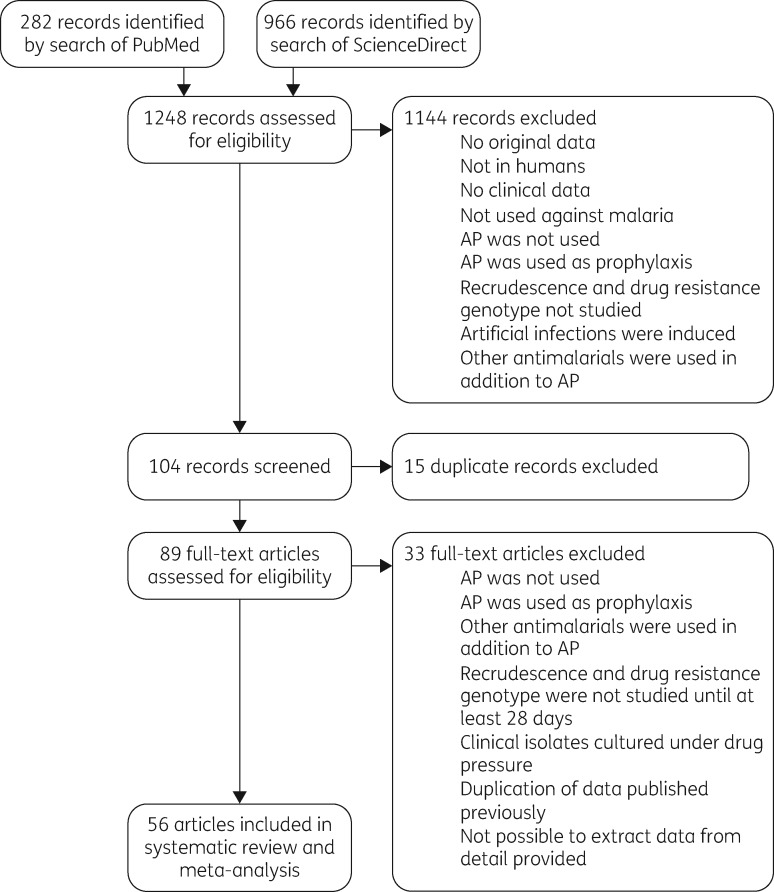
Study selection. AP, atovaquone/proguanil.

A total of 282 records were returned using PubMed and 966 using ScienceDirect (Figure 2). The 1248 records were assessed for eligibility, using title, or title and abstract, and 1144 records were excluded at this point, as they did not meet the inclusion criteria. Of the remaining 104 records, 15 duplicate records were excluded. Full text was obtained for the remaining 89 records and assessed for eligibility. Of these, 33 were excluded as they did not meet the inclusion criteria. Thus, 56 articles met the inclusion criteria for this systematic review; within these, 20 included case reports, 29 included group studies and 15 included *in vitro/ex vivo* data. The case reports and group studies were included in the meta-analysis.

The 29 group studies (Table 1) consisted of 27 with eligible data for atovaquone/proguanil treatment of *P. falciparum* infection and single studies with eligible data for atovaquone/proguanil treatment of *Plasmodium vivax* infection and *Plasmodium ovale* spp. and *Plasmodium malariae* infection. Together, the 27 *P. falciparum* studies began with 1960 patients, of whom 1695 were treated and followed up to 28 days (86.5%). A total of 1640 patients were successfully treated up to 28 days, 83.7% of the 1960 original patients and 96.8% of the 1695 treated and followed-up patients. The one *P. vivax* study began with 25 patients, of whom 19 were treated and followed up to 28 days (76%). Five patients were successfully treated up to 28 days, 20% of the original 25 patients, and 26.3% of the treated and followed up patients. The one study of *P. ovale* spp. and *P. malariae* began with six patients and all were successfully treated up to 28 days.

**Table 1.** Characteristics of group studies

Paper	Species of *Plasmodium*	Country of infection	Country of diagnosis/ treatment	Period of study	Type of study	Number of patients with ITT with atovaquone/ proguanil	Number of patients assessed at day 28	Number of patients cured at day 28	Percentage attendance	Percentage treatment success (ITT population)	Percentage treatment success (PP population)
Anabwani *et al.* 1999^30^	*P. falciparum*	Kenya	Kenya	1994	RCT	84	81	76	96.4	90.5	93.8
Borrmann *et al.* 2003^31^	*P. falciparum*	Gabon	Gabon	1999–2000	RCT	100	92	87	92	87	94.6
Bouchard *et al.* 2000^32^	*P. falciparum*	Worldwide	France	1994–95	RCT	25	21	21	84	84	100
Bustos *et al.* 1999^33^^a^	*P. falciparum*	Philippines	Philippines	1994–95	RCT	55	54	54	98	98.2	100
Carrasquilla *et al.* 2012^17^	*P. falciparum*	Columbia	Columbia	2007–08	RCT	53	53	52	100	98.1	98.1
de Alencar *et al.* 1997^34^	*P. falciparum*	Brazil	Brazil	1995–96	RCT	88	73	72	83	81.8	98.6
Gürkov *et al.* 2008^35^	*P. falciparum*	Ethiopia	Ethiopia	2006	RCT	32	30	28	93.8	87.5	93.3
Giao *et al.* 2004^36^	*P. falciparum*	Vietnam	Vietnam	2001–02	RCT	81	77	73	95.1	90.1	94.8
Llanos-Cuentas *et al.* 2001^37^	*P. falciparum*	Peru	Peru	1995–96	RCT	20	19	19	95	95	100
Looareesuwan *et al.* 1999^38^	*P. falciparum*	Thailand	Thailand	1993–94	RCT	91	79	79	86.8	86.8	100
Mulenga *et al.* 1999^39^	*P. falciparum*	Zambia	Zambia	1993–94	RCT	82	80	80	97.6	97.6	100
Mulenga *et al.* 2006^21^	*P. falciparum*	Zambia	Zambia	2000–02	RCT	128	97	92	75.8	71.9	94.8
Radloff *et al.* 1996^40^	*P. falciparum*	Gabon	Gabon	1994–95	RCT	71	63	62	88.7	87.3	98.4
Tahar *et al.* 2014^41^	*P. falciparum*	Cameroon	Cameroon	2008–09	RCT	168	156	140	92.9	83.3	89.7
				**total RCT**		**1078**	**975**	**935**			
						**weighted average (95% CI)**^b^			**92.5 (88.4, 95.8)**	**89.2 (84.7, 93)**	**97.6 (95.4, 99.2)**
Blonde *et al.* 2007^42^	*P. falciparum*	Africa	France	2004–05	Obs	48	15^d^	15	31.3	31.3	100
Boggild *et al.* 2009^43^	*P. falciparum*	Thailand	Thailand	2004–05	Obs^c^	70	68	67	97.1	95.7	98.5
Bouchard *et al.* 2012^44^	*P. falciparum*	Worldwide	Europe	2003–09	Obs	253	194	191	76.7	75.5	98.5
Chih *et al.* 2006^45^	*P. falciparum*	Africa	Australia	2003–05	Obs	52	19^d^	19	36.5	36.5	100
Gay *et al.* 1997^46^	*P. falciparum*	Worldwide	Philippines France	1993–95	Obs^c^	18	18	18	100	100	100
Grynberg *et al.* 2015^47^	*P. falciparum*	Worldwide	Israel	2001–13	Obs	44	44	38	100	86.4	86.4
Krudsood *et al.* 2007^48^	*P. falciparum*	Thailand	Thailand	2004–05	Obs	140	137	134	97.9	95.7	97.8
Lacy *et al.* 2002^49^	*P. falciparum*	Indonesia	Indonesia	1999–2000	Obs	19	19	18	100	94.7	94.7
Malvy *et al.* 2002^50^	*P. falciparum*	Worldwide	France	1999–2001	Obs	112	112	112	100	100	100
Na-Bangchang *et al.* 2005^51^	*P. falciparum*	Thailand Zambia	Thailand Zambia	2000–01	Obs	26	22	22	84.6	84.6	100
Sabchareon *et al.* 1998^52^	*P. falciparum*	Thailand	Thailand	1994–95	Obs	32	26	26	81.3	81.3	100
Tahar *et al.* 2013^53^	*P. falciparum*	Cameroon	Cameroon	2008–09	Obs	18	18	17	100	94.4	94.4
Thybo *et al.* 2004^54^	*P. falciparum*	Africa	Denmark	1999–2000	Obs	50	28	28	56	56	100
				**total Obs**		**882**	**720**	**705**			
						**weighted average (95% CI)**^b^			**87.6 (73.8, 97.1)**	**83.4 (69.7, 93.8)**	**99.1 (97.4, 99.97)**
Looareesuwan *et al.* 1996^55^	*P. vivax*	Thailand	Thailand	1990–93	Obs	25	19	5	76	20	26.3
Radloff *et al.* 1996^56^	*P. ovale* spp.	Gabon	Gabon	1995	Obs	3	3	3	100	100	100
	*P. malariae*					3	3	3	100	100	100

Obs, observational study.

a Atovaquone/proguanil data from this paper are included in the RCT section, but further analysis including data for the comparator antimalarial treatments was not undertaken for the following reason. Participants were originally randomized to atovaquone/proguanil and chloroquine, but a low cure rate for the latter resulted in a protocol amendment to include sulfadoxine/pyrimethamine. However, at the time of this change, participants in the atovaquone/proguanil arm were not separated to allow direct comparison.

b Weighted averages were calculated taking into account both population size and heterogeneity.

c Data are from an RCT, but either the study was not designed to test the efficacy of atovaquone/proguanil (or another antimalarial with atovaquone/proguanil as the control) or the trial data are not described.

d Denominator excludes patients with mixed infections or those receiving non-atovaquone/proguanil treatments (<15% of the total for each study). Denominator would increase if these patients were included, but the overall cure rates would remain unchanged at 100%.

Of note, only 14 of the studies were RCT designed to test the efficacy of atovaquone/proguanil or used atovaquone/proguanil as a control treatment and participants of these made up only 55% of the total participants included here. Most of the studies from which these data were gathered, including the RCT, were of low methodological quality, being small and having between 18 and 253 participants receiving atovaquone/proguanil. Risk of bias during selection was determined to be unclear in 10 of 14 RCT group studies, as methods for randomization and concealment of allocation were unclear (Table 2). Risk of bias during performance was determined to be high in 13 of 14 studies, as blinding of participants and researchers was used in only one study. Risk of detection bias was determined to be unclear in all but one RCT study, as allocated interventions were not blinded. Risk of bias due to a high rate of attrition (<10%, low; between 10% and 20%, medium; >20% high) or patients withdrawn from the trial without explanation was high in only one RCT study. Risk of bias due to selective reporting was low to medium in all studies as 28 day cure rate was defined as either a primary (low) or secondary (medium) outcome in all cases. Another potential bias was that 11 of the 14 RCT studies were carried out by, funded by or supported by GlaxoSmithKline or its preceding companies Glaxo Wellcome and Wellcome Research Laboratories.

**Table 2.** Risk of bias in RCT

Paper	Type of bias						
	selection		performance	detection	attrition	reporting	other
	RSG	AC					
Anabwani *et al.* 1999^30^	unclear	unclear	high	unclear	low	low	unclear
Borrmann *et al.* 2003^31^	low	low	high	unclear	medium	low	unclear
Bouchard *et al.* 2000^32^	unclear	unclear	high	unclear	medium	low	unclear
Bustos *et al.* 1999^33^	unclear	unclear	high	unclear	low	low	unclear
Carrasquilla *et al.* 2012^17^	unclear	unclear	high	low	low	medium	low
de Alencar *et al.* 1997^34^	unclear	unclear	high	unclear	medium	medium	unclear
Gürkov *et al.* 2008^35^	unclear	unclear	high	unclear	low	medium	low
Giao *et al.* 2004^36^	low	low	high	unclear	low	medium	unclear
Llanos-Cuentas *et al.* 2001^37^	unclear	unclear	high	unclear	low	low	unclear
Looareesuwan *et al.* 1999^38^	unclear	unclear	high	unclear	medium	low	unclear
Mulenga *et al.* 1999^39^	unclear	unclear	high	unclear	low	low	unclear
Mulenga *et al.* 2006^21^	low	unclear	low	unclear	high	low	unclear
Radloff *et al.* 1996^40^	low	unclear	high	unclear	medium	medium	unclear
Tahar *et al.* 2014^41^	unclear	unclear	high	unclear	low	medium	low

RSG, random sequence generation; AC, allocation concealment.

High-quality data for the efficacy of atovaquone/proguanil are scarce, but provide estimates of treatment success in RCT group studies of between 89% and 98% for *P. falciparum* malaria (Table 1; weighted averages based on population size and heterogeneity), between 20% and 26.3% for *P. vivax* malaria (from one study) and 100% (in three patients each) for *P. malariae* and *P. ovale* spp. malaria.

**Figure 3.**
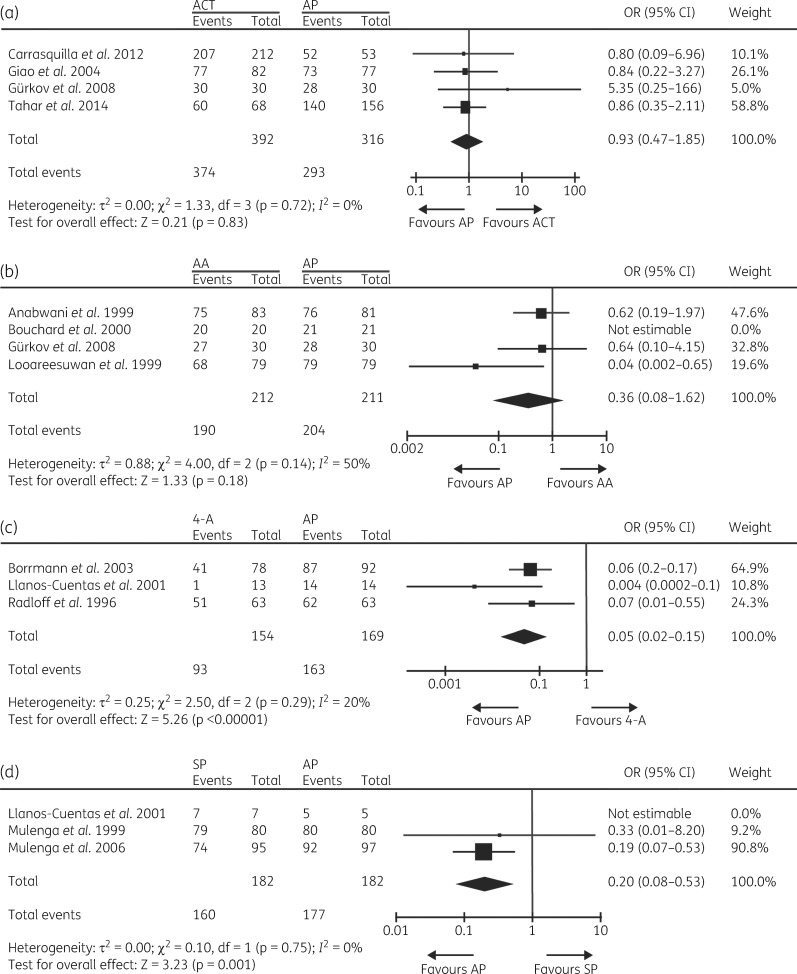
Forest plots for the relative treatment successes at day 28 of patients treated with atovaquone/proguanil (AP) or (a) ACT, (b) amino alcohols (AA), (c) 4-aminoquinolines (4-A) or (d) sulfadoxine/pyrimethamine (SP).

Comparator antimalarial treatments (with number of times trialled in parentheses) were chloroquine (two), amodiaquine (two), sulfadoxine/pyrimethamine (three), chloroquine/sulfadoxine/pyrimethamine (one), quinine (one), quinine/tetracycline (one), halofantrine (two), mefloquine (one), and the artemisinin-based combination therapies (ACT), artemether/lumefantrine (two), artesunate/mefloquine (one), artesunate/amodiaquine (one) and dihydroartemisinin/piperaquine/trimethoprim/primaquine (one). Nine of the 14 RCT presented here were analysed in a previous Cochrane Library systematic review from 2005.^16^ Subsequent RCT involving atovaquone/proguanil have used ACT predominantly as the comparator treatment(s). Given the diversity of treatments used in the trials and to allow results to be generalized to a larger population, trial data involving ACT, 4-aminoquinolines (chloroquine and amodiaquine) and amino alcohols (mefloquine, halofantrine and quinine), were grouped for a meta-analysis (Table S1). Sulfadoxine/pyrimethamine was analysed alone. The analysis indicates that there is no significant difference (*P *=* *0.83) in treatment success between the use of atovaquone/proguanil and ACT (Figure 3a). Sensitivity analysis was consistent with this outcome (Table S2). Given the grouped ACT in this analysis, we combined the data for two different ACT in one three-arm study.^17^ However, analysing each arm separately did not change the outcome of the analysis (Table S2). Analysis of atovaquone/proguanil versus the amino alcohols group (Figure 3b) indicates that treatment success with atovaquone/proguanil is not significantly more effective (*P *=* *0.18) and statistical significance was maintained for the majority of scenarios during sensitivity analysis (Table S2). As previously reported individually for amodiaquine and chloroquine,^16^ meta-analysis of the three trials that used atovaquone/proguanil versus 4-aminoquinolines (Figure 3c) suggested that atovaquone/proguanil is more effective than 4-aminoquinolines (*P *<* *0.00001) and the sensitivity analysis was predominantly consistent with this outcome (Table S2). This can be explained by the prevalence of mutations in *pfcrt* and *pfmdr1* conferring resistance to chloroquine and amodiaquine in the regions of study.^18–20^ Similar findings (*P *=* *0.001) emerged when analysing atovaquone/proguanil versus sulfadoxine/pyrimethamine (Figure 3d and Table S2). This can be explained by the increasing development of sulfadoxine/pyrimethamine resistance over time between the two studies undertaken in Zambia.^21^^,^^39^

Eligible data on *in vitro*/*ex vivo* clinical isolates exposed to atovaquone were available in 15 papers (Table 3). The amount of data and the level of detail available did not allow further mathematical syntheses, but the data can be used to hypothesize about what the *in vitro*/*ex vivo* phenotype of atovaquone resistance might be. All *P. falciparum* isolates with the WT Y amino acid at codon 268 have an atovaquone IC_50_ ≤28 nM, with the majority <10 nM. All single isolates with N, C or S at 268 have IC_50_ values between 20.5 and 17 000 nM. A further four isolates with S at 268 were reported to have a median (IQR) IC_50_ value of 5.7 nM (1.7–1216).^22^ Isolates with mixed genotypes were susceptible to atovaquone *in vitro*, with median IC_50_ values between 4.7 and 5 nM. Isolates of unknown genotype ranged in IC_50_ values from low nanomolar to low micromolar. The 38 *P. vivax* isolates had a pooled mean IC_50_ value of 29.4 nM.^23^

**Table 3.** Characteristics of *in vitro/ex vivo* studies

Paper	Species of *Plasmodium*	Country of infection	Country of diagnosis/ treatment	Period of study^a^	Number of isolates	Atovaquone IC_50_ (nM)	Dispersion (nM)	Codon 268
Basco 2003^57^	*P. falciparum*	Cameroon	Cameroon	2001–02	37	0.58 geometric mean	0.27–2.2 range	Y
Durand *et al.* 2008^58^	*P. falciparum*	DRC	France	2007	1^b^	10	not stated	Y
Fivelman *et al.* 2002^11^	*P. falciparum*	Nigeria	UK	*2002*	1^c^	1888 mean	107 SD	N
Gay *et al.* 1997^46^	*P. falciparum*	worldwide	The Philippines, France	1993–95	96	1.4 median	5.5 90^th^ percentile	–
Ingasia *et al.* 2015^22^	*P. falciparum*	Kenya	Kenya	2008–12	143	3 median	1–6.9 IQR	Y
					4	5.7 median	1.7–1216 IQR	S
					74	4.7 median	2.2–11.1 IQR	Y/S
					6	5 median	2–11.8 IQR	Y/S/N
Khositruithikul *et al.* 2008^59^	*P. falciparum*	Thailand	Thailand	1998–2005	83	3.4 mean	1.6 SD	Y
							0.83–6.81 range	
Legrand *et al.* 2007^60^	*P. falciparum*	French Guiana	French Guiana	2005	1^b^	1.6	not stated	Y
					1^c^	20.5	not stated	S
Looareesuwan *et al.* 1996^55^	*P. falciparum*	Thailand	Thailand	1990–93	12^b^	9 mean	not stated	–
					NS	13 486 mean	not stated	–
					3^c^	10.4 mean	not stated	–
					3^d^	3.3 mean	not stated	–
Lütgendorf *et al.* 2006^61^	*P. falciparum*	Thailand	Thailand	2000	37^b^	3.2	not stated	–
Musset *et al.* 2006^62^	*P. falciparum*	worldwide	France	1999–2004	477	1.79 geometric mean, 2 median^e^	0.1–28 range	Y
					1^c^	8230	not stated	S
Musset *et al.* 2006^12^	*P. falciparum*	W. Africa	France	2003–05	1^c^	9.89	not stated	Y
					1^c^	1.49	not stated	Y
					1^c^	7.87	not stated	Y
					1^c^	17 000	not stated	C
					1^c^	8230	not stated	S
					1^c^	10 400	not stated	S
Savini *et al.* 2008^63^	*P. falciparum*	Comoros	France	*2008*	1^b^	2.9	not stated	Y
					1^c^	390	not stated	S
Tahar *et al.* 2014^41^	*P. falciparum*	Cameroon	Cameroon	2008–09	55^b^	1.32 geometric mean	1.06–1.65 95% CI	Y
							0.184–5.30 range	
Treiber *et al.* 2011^23^	*P. vivax*	Thailand	Thailand	2008	38	29.4 mean	not stated	–
van Vugt *et al.* 2002^64^	*P. falciparum*	Thailand	Thailand	1998–2000	39^b^	2.21 median	0.11–17.8 range	–
					10^c^	2.86 median	0.84–38.9 range	–

NS, recurrence after atovaquone treatment alone – although number not stated.

a Where not given, the year of publication is given in italics.

b Pretreatment.

c Recurrence after atovaquone/proguanil treatment.

d Pretreatment isolates from ^c^.

e Means include the data from the isolate taken after recurrence after atovaquone/proguanil treatment.

Data for case reports were available from 20 papers for 36 individuals (Table 4). Thirty-three of the cases were of *P. falciparum* infection and there was one case each of *P. malariae*, *P. ovale* spp. and *P. vivax* infection. Variables have been summarized, with means, standard deviations (SD), medians and IQR for continuous or count data and proportions for categorical or binary data types (Table S3). Data for pretreatment parasitaemia (baseline), parasitaemia at treatment failure/recrudescence and genotype were not available for non-*falciparum* infections and so these species were not included in subsequent analyses.

**Table 4.** Characteristics of case reports

Paper	Species of *Plasmodium*	Country of infection	Country of diagnosis/ treatment	Period of study^a^	Pretreatment parasitaemia (%)	Codon 268 pretreatment^b^	Days until symptomatic	Days until parasitological diagnosis	Minimum days until recrudescence	Parasitaemia at recrudescence (%)	Codon 268 post- treatment^b^
Blossom *et al.* 2005^65^	*P. vivax*	Zambia	USA	2002	–	–	21	21	21	–	–
Contentin *et al.* 2011^66^	*P. falciparum*	Guinea	France	*2011*	7	–	20	20	20	1.7	–
David *et al.* 2003^67^	*P. falciparum*	Cameroon	Denmark	2002	1	–	21	21	21	2.5	–
Durand *et al.* 2008^58^	*P. falciparum*	DRC	France	2007	1.6	Y**	–	28	28	0.001	Y**
Färnert *et al.* 2003^10^	*P. falciparum*	Ivory Coast	Sweden	2000	1	Y*	2	2	2	4	Y*
					0.5	S*	28	28	28	1.6	S**
Fivelman *et al.* 2002^11^	*P. falciparum*	Nigeria	UK	*2002*	1.5	–	28	33	28	<1	N
Forestier *et al.* 2011^68^	*P. falciparum*	Cameroon	France	2009	2	–	21	21	21	3	–
Koch *et al.* 2007^69^	*P. falciparum*	Ghana	Germany	*2007*	1	–	4	4	4	<1	–
Kuhn *et al.* 2005^70^	*P. falciparum*	Sierra Leone	Canada	*2005*	–	Y**	19	–	19	–	S**
Legrand *et al.* 2007^60^	*P. falciparum*	French Guiana	French Guiana	2005	–	Y**	–	24	24	–	S**
Müller-Stöver *et al.* 2007^71^	*P. malariae*	Nigeria	Germany	*2007*	–	–	98	98	98	–	–
Musset *et al.* 2006^12^	*P. falciparum*	W. Africa	France	2003–05	0.002	Y*	3	3	3	0.5	Y*
					0.3	Y**	–	7	7	1	Y**
					0.007	Y**	11	11	11	0.75	Y**
					0.35	Y**	22	22	22	0.47	C**
					13	Y**	26	26	26	5	S**
					4	Y**	26	26	26	5	C**
					0.15	Y**	39	39	39	0.25	S**
					0.2	Y*	3	3	3	1.1	Y*
					2.8	Y**	–	28	28	1.5	S**
Oswald *et al.* 2007^72^	*P. ovale* spp.	Mozambique	USA	*2007*	–	–	31	45	31	–	–
Perry *et al.* 2009^73^	*P. falciparum*	India, Nepal	Canada	2007	3.4	–	18	34	18	2	C
Plucinski *et al.* 2014^74^	*P. falciparum*	Nigeria	USA	2012–13	<5	Y**	31	34	31	3	S**
Rose *et al.* 2008^75^	*P. falciparum*	Mozambique	Canada	2006	1.2	–	–	33	33	3.2	S
Savini *et al.* 2008^63^	*P. falciparum*	Comoros	France	*2008*	0.5	Y**	23	23	23	1.3	S**
Schwartz *et al.* 2003^76^	*P. falciparum*	Kenya	Israel	2002	3	Y**	30	30	30	–	S**
Sutherland *et al.* 2008^27^	*P. falciparum*	Africa	Africa, UK, Switzerland	2004–08	–	–	–	42	42	1.1	C
					1	–	2	2	2	4	Y
					2.5	–	3	3	3	0.1	Y
					0.1	–	23	25	23	0.3	S
					–	–	–	4	4	1	Y
					–	–	–	21	21	0.2	C
					<0.1	Y	26	26	26	<0.1	C
					–	–	32	32	32	3	C
Wichmann *et al.* 2004^77^	*P. falciparum*	DRC	Germany	*2004*	0.1	Y	19	19	19	0.01	Y

a Where not given, the year of publication is given in italics.

b Where given,

* *PfDHFR* S108, N51, C59 and

** *PfDHFR* S108N, N51I, C59R.

**Figure 4.**
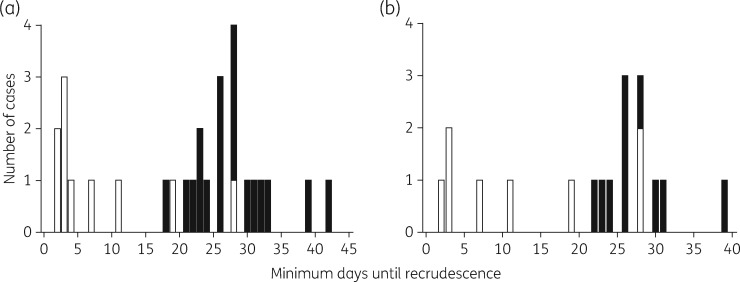
Relationship between the number of days until recrudescence of malaria infection and the status of codon 268 in *PfCYTb*. Numbers of cases of patients infected with *P. falciparum* parasites (a) with (white bars) or without (black bars) mutation at codon 268 in *PfCYTb* at the time of recrudescence and (b) with (white bars) or without (black bars) a change at codon 268 in *PfCYTb* between the initial infection and the time of recrudescence.

A raw data plot, Figure 4(a), presents the minimum number of days to recrudescence of infection after atovaquone/proguanil treatment, which takes into account the onset of symptoms if prior to parasitological diagnosis, versus the absence or presence of mutation (Y268S/C/N) in *PfCYTb* at the time of recrudescence. This suggests that distributions may differ across groups by mutation (confirmed by a preliminary Kruskal–Wallis test; *P *<* *0.001). In a subset of parasite isolates it was possible to define if there had been a change in codon 268 following treatment. A raw data plot of the minimum number of days to recrudescence versus this dataset suggested distributions may differ by codon 268 change (*P *=* *0.009; Kruskal–Wallis test; Figure 4b).

**Figure 5.**
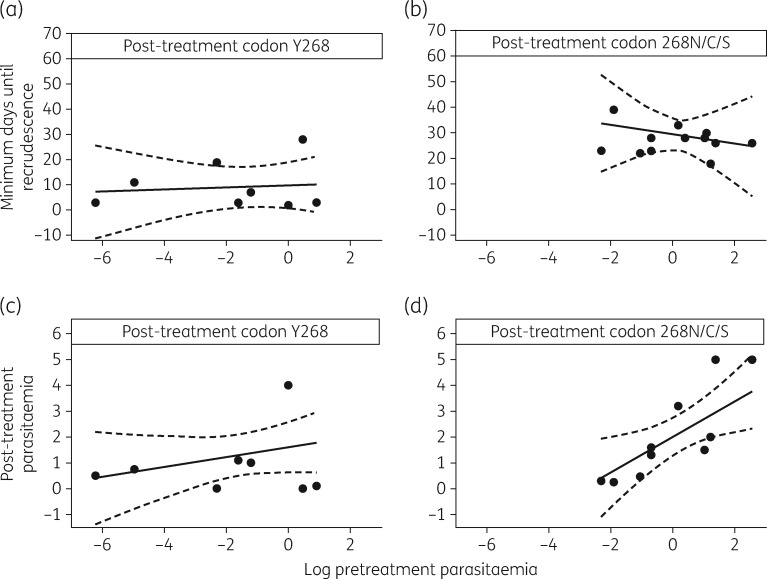
Relationship between pretreatment parasitaemia and (a and b) minimum days until recrudescence and (c and d) post-treatment parasitaemia in the absence or presence of mutation at codon 268 in *PfCYTb*. Complete data sets (filled circles) are shown with predicted lines of fit by multiple imputation (continuous lines) and their 95% CI (broken lines).

Figure 5 presents the relationship between pretreatment parasitaemia and minimum days until recrudescence in the absence or presence of a mutation in *PfCYTb*, using an interaction model (Figure 5a and b). Analyses of the complete and observed (by multiple imputation) datasets suggest that pretreatment parasitaemia does not appear to influence the minimum days until recrudescence in general and that there is evidence that this effect is not modified by the presence of mutation in *PfCYTb* (*P *=* *0.62 and 0.87, respectively; Table S4). However, according to complete data analysis, there is evidence (*P *<* *0.001; Table S4) that grouping (the codon 268 present post-treatment) is a statistically significant predictor of the minimum days until recrudescence and the evidence is further supported by the observed data analysis (*P *=* *0.002; Table S4). The model predicts that patients presenting with a baseline parasitaemia of 1% will have an average minimum number of days until recrudescence of 29 (95% CI: 22, 35) days if mutation in codon 268 in *PfCYTb* is present, whilst this is 19 (95% CI: 7.3, 30) days shorter in duration if the mutation is absent. Note that although a slight departure from normality for the standardized residuals (*P *=* *0.02) was calculated, we opted for model simplicity rather than introducing another quadratic term.

Figure 5 also presents the relationship between baseline pretreatment parasitaemia and parasitaemia at recrudescence (post-treatment parasitaemia) in the absence or presence of a mutation in *PfCYTb*, using an interaction model (Figure 5c and d). Analyses of the complete and observed datasets suggest that baseline parasitaemia (on a log scale) increases slightly and linearly with parasitaemia at recrudescence of infection (*P *=* *0.004 and 0.029, respectively; Table S5). Furthermore, analysis of the complete dataset suggests that the level of increase differs by grouping using codon 268 presence post-treatment, although this effect no longer holds when observed data analysis has been implemented (*P *=* *0.04 versus *P *=* *0.217; Table S5). Note that the two settings do not exhibit massive differences in estimates and their precisions. Here, the model predicts that patients presenting with a baseline parasitaemia of 1% (geometric mean, which coincides with the median; Table S5) will have an average post-treatment parasitaemia of 2.0% (95% CI: 1.2%, 2.8%) if a mutation in codon 268 in *PfCYTb* is present.

Additional analyses to incorporate pretreatment parasitaemia interval values as <0.01 and <5 (Table 4), using scenarios in which these values were ‘1’, their upper limit, ‘2’, half the interval values and ‘3’, a 10th of the value, provided no substantial quantitative changes in the above estimates presented and their precision and no qualitative changes to the conclusion (Table S6 and Table S7).

## Discussion

Atovaquone/proguanil was developed as a combination therapy when early clinical studies showed that atovaquone as a single agent was associated with recrudescence of highly atovaquone-resistant infections in ∼30% of patients.^24^*In vitro* evidence of synergy with proguanil prompted development of this combination, whose initial high cost precluded widespread use. As generic formulations of atovaquone/proguanil reduce costs, and as TFACT emerge, atovaquone/proguanil is one of the few non-ACT combinations registered for management of malaria. Determining its overall efficacy and identifying markers that predict treatment failures is important for policymakers in public health.

To carry out the widest scrutiny of evidence on the efficacy of atovaquone/proguanil, we included two broad types of studies. The first type (summarized in Table 1) describes efficacy of atovaquone/proguanil in the treatment of malaria often (in just over 50% of cases) in the context of an RCT. The quality of these types of studies is relatively low for several reasons associated with potentials for bias (Table 2). The second more mechanistic analysis of atovaquone/proguanil’s efficacy (summarized in Tables 3 and 4) included review of *in vitro* susceptibility analysis of parasites, where available, and detailed analysis of individual case reports of treatment failures and their association with parasitaemia and mutation in *PfCYTb*. These latter reports are often richer in data and provide insights that complement findings from larger studies.

While datasets were small and associated with potential bias (and thus requiring cautious interpretation), the overall efficacy of atovaquone/proguanil expressed as a weighted average based on study population sizes and heterogeneity is 89% and 83% in ITT analyses of RCT and observational studies, respectively, and is 98% and 99% in PP analyses. This is a reassuringly acceptable level of efficacy and to date there are no indications of treatment failures becoming associated with particular geographical areas that would preclude atovaquone/proguanil use to treat travellers or prevent infections from such areas. Furthermore, meta-analysis suggests that atovaquone/proguanil treatment success is equivalent to the use of ACT and amino alcohols and better than 4-aminoquinolines and sulfadoxine/pyrimethamine, although caution is required in some cases due to the grouping of different antimalarials within a class. This extends findings from a prior meta-analysis that concluded that atovaquone/proguanil is more effective than chloroquine, amodiaquine and mefloquine.^16^ This general reassurance is important particularly in light of complications that are being associated with the use of mefloquine and that have been reviewed recently in a UK House of Commons Defence Committee report on mefloquine’s use in military personnel.^25^ Doxycycline and atovaquone/proguanil remain as the only alternatives to mefloquine recommended for antimalarial prophylaxis.^5^ While atovaquone/proguanil is considered safe, it has been reported that safety data are relatively sparse and would benefit from further large trials.^16^ The safety of atovaquone/proguanil was not studied here.

The *in vitro* phenotypic assays for atovaquone susceptibility and its relationship to target genotype suggest that WT amino acid (Y268) is uniformly associated with susceptibility. The threshold for defining susceptibility is an IC_50_ value ≤28 nM, with most isolates in different studies having IC_50_ values <10 nM. Although the aggregated IC_50_ values for *P. vivax* were 29 nM, it is unlikely that this slightly higher value compared with *P. falciparum* susceptibility contributed to the higher treatment failure rates as these are most likely due to relapse because of the non-susceptibility of hypnozoite stages found in the liver to atovaquone/proguanil.^26^

Analysis of individual case reports and the dynamics of recrudescing infection highlight further interesting findings. The presence or appearance of mutation (Y268S/C/N) in *PfCYTb* is strongly associated with a late recrudescing infection (Figures 4 and 5) where late onset of symptoms or parasitological recrudescence (whichever is earlier, which we have defined as minimum days to recrudescence here) is on average 29 days (95% CI: 22, 35) after treatment has commenced. This is in accord with a previous estimate of the mean time to recrudescence of parasites carrying the Y268C mutation of 28 days (95% CI: 23.0, 33.0).^27^ Understanding the mechanisms that account for the length of time until recrudescence is worthy of further investigation. One possible factor underlying this phenotype is a loss of parasite fitness due to mutation. This has been reported previously, using *in vitro* growth assays, for atovaquone-resistant parasites carrying *PfCYTb* mutations, though not at position 268.^28^ Our data suggest that patients should be monitored for up to 42 days. Late recrudescence in these cases should always be treated with an alternative antimalarial treatment regimen.

A recent report has demonstrated that mutations in *P. berghei CYTb* are invariably lethal to the parasite during transmission in the mosquito vector.^13^ This finding lends weight to the hypothesis that *PfCYTb* mutations may not be able to spread within a population. If true, this would preclude the requirement to monitor for these mutations in endemic areas. The available data are in general agreement with this, as codon 268 mutations are very rarely observed in parasites from patients that suffer later recrudescence, prior to drug pressure (Table 4) and no geographical foci of atovaquone/proguanil treatment failure or *PfCYTb* mutations have been reported. However, this does not preclude the spread of *PfCYTb* mutations carried by parasite sub-populations, where the mutation cannot be detected by conventional means, or the spread of parasites with permissive genetic backgrounds that favour *PfCYTb* mutation following drug pressure. Our findings also identify the need for further characterization of the genetic backgrounds of parasites in patients experiencing early recrudescence. These studies should aim to determine the mechanism of this high-grade resistance as well as identifying associated markers, although other factors that may cause or contribute to the phenotype of early treatment failure will need to be considered carefully (e.g. non-compliance to treatment, use of substandard or counterfeit medications, poor absorption or metabolism of the medication by the patient).

While not considered in detail, it is worth noting that there are 17 case reports that provide molecular markers for cycloguanil resistance, the triple *PfDHFR* mutation S108N, N51I, C59R (Table 4). Only 4 of 17 infections carried parasites with sensitive genotypes at first presentation. One of these four infections recrudesced with parasites carrying a resistant genotype, leaving three infections caused by parasites with *PfDHFR*-inhibitor sensitive genotypes post-treatment. Interestingly, all parasites defined as recrudescing by day 3 (Table 4) carried *PfDHFR* sensitive genotypes, suggesting that cycloguanil did not contribute to failure. All later treatment failures (from day 7) were caused by parasites carrying genotypes associated with resistance to cycloguanil. Therefore, atovaquone/proguanil treatment failures from day 7 onwards are most likely to be caused by parasites that are already resistant to cycloguanil.

After our database search was closed, an additional series of case reports that was not picked up was identified independently.^29^ These six cases were of patients who had recrudesced more than once after atovaquone/proguanil treatment and in all cases time to recrudescence was ≥19 days. In five cases where the post-treatment genotype of *PfCYTb* was available, it was of the 268C/S mutation. In four of six patients with second recrudescences, the time to recrudescence was ≥20 days and all four genotypes bore mutant variants at position 268. These observations suggest that the proguanil component of atovaquone/proguanil has sufficient antimalarial efficacy to suppress parasitaemias for 2–3 weeks and that the dynamics of late treatment failure are consistent with absence of atovaquone efficacy. These cases were incorporated into a secondary analysis of the case reports. Findings with regard to the relationship between pretreatment parasitaemia and minimum days until recrudescence in the absence or presence of a mutation in *PfCYTb* are consistent with those presented in Table S8. 

Overall, atovaquone/proguanil therapy is comparable in efficacy to ACT used in treating uncomplicated malaria. Detailed genotype–phenotype analysis in this systematic review has illustrated several new findings. There are differences between early and late treatment failures because mutations in the target conferring resistance to atovaquone are identified most commonly in late and not early treatment failures. The mechanism of early treatment failure after atovaquone/proguanil treatment needs further investigation. Recent evidence is also reassuring that spread of the 268 mutations conferring atovaquone resistance may be limited by poor transmissibility in the insect stages of *P. falciparum* infections.

## Supplementary Material

Supplementary Data
