# Supplementary material for: Clinical implications of Plasmodium resistance to atovaquone/proguanil: a systematic review and meta-analysis
Source: J Antimicrob Chemother. 2017 Dec 11;73(3):581–95. doi: 10.1093/jac/dkx431 (PMC5890752; doi:10.1093/jac/dkx431)
Supplement: Supplementary Data [file supplementary_data_dkx431.docx]

**Supplementary data**

**Supplementary Methods**

***Inclusion/exclusion criteria***

Original published data from all group studies and case reports were included where atovaquone/proguanil was used exclusively as an antimalarial treatment for natural *Plasmodium* infections. Data from all *ex vivo*/*in vitro* studies were included where atovaquone was tested alone against clinical isolates of *Plasmodium* infections. We excluded all studies where atovaquone/proguanil was used in conjunction with other antimalarials, to avoid confounding with the efficacy of atovaquone/proguanil as the sole treatment and on the *in vitro* phenotype of resistance. We included studies where other medications without antimalarial activity were used. As this is a review of efficacy and resistance, we included only studies where efficacy or resistance were studied; group studies were excluded if a 28-day follow-up period was not used, and case reports were excluded when neither treatment failure, recrudescence, or codon 268 of *PfCYTb* were reported. *Ex vivo*/*in vitro* studies were excluded when quantitative IC_50_/EC_50_ values were not published, as qualitative information did not contribute towards defining an *ex vivo*/*in vitro* resistance phenotype.

***Extracted data variables***

Where data were available, the following variables were summarised: study design, participant inclusion criteria, participant exclusion criteria, species of malaria, country of infection, country of diagnosis, the dose of atovaquone/proguanil, whether PCR-correction was carried out, the atovaquone IC_50_ value, the number of participants/isolates, the number of participants for whom 28-day follow-up was carried out, whether samples were taken before treatment or after treatment failure/recrudescence, the genotype of *PfCYTb* codon 268 before treatment and/or after treatment failure/recrudescence, parasitaemia before treatment or after treatment failure/recrudescence, the number of participants in whom treatment failed or recrudescence occurred, other genotyping of drug resistance markers, how many days between starting treatment and treatment failure/recrudescence (defined by time of recurrence of symptoms, and/or laboratory confirmation), source of funding and declared conflicts of interest by the authors. Where authors specified species, we assumed that their speciation was accurate. Where many countries were sites in a group study, we simplified the list to describe the region in which these countries are. Where individual mutations were not described but a group of mutations were, we interpreted this to mean the most common form of that collection of mutations. When time between treatment and treatment failure/recrudescence was described as approximately a week, we counted this as seven days, when it was described in hours, we rounded to the nearest day. Language in case reports was sometimes unclear when discussing days to recrudescence, and days were described as “later” rather than taken from the time of initiation of treatment. Where days to recrudescence were unclear, we tried to interpret the intended meaning, rather than use an assumption that all times were from either start or finish of treatment course. Different mutant 268 alleles (N/C/S) were cumulated into one group in the data due to the small number of cases available for analysis. The *in vitro* IC_50_ of atovaquone was used as a principal summary measure.

***Group study sensitivity analyses***

The results have been accompanied by the following sensitivity analyses. Firstly, studies with 100% treatment success in a single arm were discarded and the analyses re-run. Secondly, missing (undiagnosed) individuals between the PP and ITT placed into a series of plausible scenarios defined as: a) best case – in which the treatment favours atovaquone/proguanil: those who were not assessed in the atovaquone/proguanil group were all treatment successes and those who were not assessed in the comparator groups were all treatment failures, b) worst case – the treatment favours the comparator intervention: those who were not assessed in the atovaquone/proguanil group were all treatment failures and those who were not assessed in the comparator groups were all treatment failures, c) missing successes: all those who were not assessed were treatment successes, and d) missing failures: all those who were not assessed were treatment failures.

***Regression techniques***

The two statistical outcomes were deemed as continuous and log transformations were considered. Selection of the appropriate model was based initially on an appropriate fit (normality assumption of standardized residuals) on complete data. Observed data analyses have then been conducted using multiple chain imputation techniques and the results discussed in this context. The general assumption for missing data is that of ‘missing at random’, (i.e. any systematic difference between the missing values and the observed values can be explained by differences in observed data).^1^ Multiple imputation procedures accounted for distributional assumptions for the variables that necessitated imputation.^2^ In the presence of missing data, formal classical tools for models’ selection and goodness of fit no longer apply. Therefore, the final presented models are the result of a combination of available data, clinically relevant mechanisms, model parsimony and reasonable statistical fit based on complete data. Resulting estimates with p-values less than 0.05 are considered statistically significant.

The models suggest that log transformed baseline parasitaemia provides a better fit for all outcomes of interest. Therefore, this variable has been log transformed and centred in its mean (its geometric mean at linear scale) in all models for a unified approach and meaningful interpretation of the resulting constant in the models. Moreover, this variable has always been left in the model, regardless of its strict statistical significance (given the rather observational feature of the study).

***Supplementary Methods references***

1. Sterne JA, White IR, Carlin JB *et al.* Multiple imputation for missing data in epidemiological and clinical research: potential and pitfalls. *BMJ* 2009; **338**: b2393.

2**.** White IR, Royston P, Wood AM. Multiple imputation using chained equations: Issues and guidance for practice. *Stat Med* 2011; **30**: 377-99.

**Table S1.** Data used for meta-analysis, comparing treatment success by 28 days of 4 antimalarial groupings versus atovaquone/proguanil (AP)

| **Atovaquone/Proguanil (AP)** | |  |  |  | **Comparator** | |  |  |  |
| --- | --- | --- | --- | --- | --- | --- | --- | --- | --- |
| **Study** | **Year** | **Number of Patients with Intention to Treat with AP** | **Number of Patients Assessed at D28** | **Number of Patients Cured at D28** | **Grouping** | **Antimalarial** | **Number of Patients with Intention to Treat with AC** | **Number of Patients Assessed at D28** | **Number of Patients Cured at D28** |
| Anabwani *et al.* 1999 | 1999 | 84 | 81 | 76 | **Amino Alcohols** | Halofantrine | 84 | 83 | 75 |
| Bouchard *et al.* 2000 | 2000 | 25 | 21 | 21 | **(AA)** | Halofantrine | 23 | 20 | 20 |
| Gurkov *et al.* 2008 | 2008 | 32 | 30 | 28 |  | Quinine | 35 | 30 | 27 |
| Looareesuwan *et al.* 1999 | 1999 | 91 | 79 | 79 |  | Mefloquine | 91 | 79 | 68 |
|  |  |  |  |  |  |  |  |  |  |
| Borrmann *et al.* 2003 | 2003 | 100 | 92 | 87 | **4-Aminoquinolines** | Amodiaquine | 100 | 78 | 41 |
| Llanos-Cuentas *et al.* 2001* | 2001 | 15 | 14 | 14 | **(4-A)** | Chloroquine | 14 | 13 | 1 |
| Radloff *et al.* 1996 | 1996 | 71 | 63 | 62 |  | Amodiaquine | 71 | 63 | 51 |
|  |  |  |  |  |  |  |  |  |  |
| Llanos-Cuentas *et al.* 2001* | 2001 | 5 | 5 | 5 | **Sulphadoxine/** | SP | 9 | 7 | 7 |
| Mulenga *et al.* 1999 | 1999 | 82 | 80 | 80 | **Pyrimethamine** | SP | 81 | 80 | 79 |
| Mulenga *et al.* 2006 | 2006 | 128 | 97 | 92 | **(SP)** | SP | 127 | 95 | 74 |
|  |  |  |  |  |  |  |  |  |  |
| Carrasquilla *et al.* 2012** | 2012 | 53 | 53 | 52 | **Artemisinin-Based** | Art+Lum | 159 | 159 | 155 |
|  |  |  |  |  | **Combination** | Ars+Mef | 53 | 53 | 52 |
| Giao *et al.* 2004 | 2004 | 81 | 77 | 73 | **Therapies** | DHA+Pip+Trim+PQ, CV8 | 84 | 82 | 77 |
| Gurkov *et al.* 2008 | 2008 | 32 | 30 | 28 | **(ACT)** | Art+Lum | 30 | 30 | 30 |
| Tahar *et al.* 2014*** | 2014 | 168 | 156 | 140 |  | Ars+Amodiaquine | 70 | 68 | 60 |

*This 2 arm study was undertaken in 2 phases (AP versus chloroquine and then AP versus SP, since chloroquine was not found to be effective), where the AP data have been separated at the point of protocol amendment

**ACT data from this 3 arm study was combined for the main analysis

***Two AP arms from this 4 arm study were combined. This study also included a 100 patient arm treated with Ars+Amodiaquine+AP - this is also an ACT but has been excluded as it contains AP also

**Table S2.** Meta-analysis summary data, including sensitivity analysis, for comparing treatment success by 28 days of 4 antimalarial groupings versus atovaquone/proguanil (AP)

| Intervention | Sensitivity analysis | Number of studies analysed* | Pooled estimate - OR  Intervention vs AP | 95%CI | OR = 1 test  (p-value) | Heterogeneity test  (p-value) |
| --- | --- | --- | --- | --- | --- | --- |
| Artemisinin-Based | Original** | 4 | 0.93 | 0.47, 1.85 | 0.83 | 0.72 |
| Combination | Original** | 3 | 0.85 | 0.42, 1.72 | 0.65 | 0.99 |
| Therapies | Best | 4 | 0.69 | 0.36, 1.33 | 0.26 | 0.62 |
| (ACTs) | Best | 3 | 0.63 | 0.32, 1.23 | 0.17 | 0.97 |
|  | Worst | 4 | 1.65 | 0.87, 3.10 | 0.12 | 0.61 |
|  | Worst | 3 | 1.51 | 0.79, 2.89 | 0.21 | 0.82 |
|  | Missing-successes | 4 | 0.89 | 0.45, 1.78 | 0.75 | 0.73 |
|  | Missing-successes | 3 | 0.82 | 0.40, 1.65 | 0.57 | 0.99 |
|  | Missing-failures | 4 | 1.27 | 0.70, 2.29 | 0.43 | 0.56 |
|  | Missing-failures | 3 | 1.16 | 0.64, 2.13 | 0.62 | 0.94 |
|  | Original*** | 4 | 0.95 | 0.47, 1.92 | 0.88 | 0.73 |
|  | Original*** | 3 | 0.86 | 0.42, 1.78 | 0.69 | 0.99 |
|  | Original**** | 4 | 0.92 | 0.46, 1.85 | 0.82 | 0.72 |
|  | Original**** | 3 | 0.84 | 0.41, 1.71 | 0.63 | 0.99 |
| Amino Alcohols | Original | 3 | 0.36 | 0.08, 1.62 | 0.18 | 0.14 |
| (AAs) | Original | 2 | 0.62 | 0.23. 1.67 | 0.35 | 0.97 |
|  | Best | 4 | 0.17 | 0.04, 0.77 | **0.02** | 0.07 |
|  | Best | 2 | 0.40 | 0.16, 1.02 | 0.05 | 0.40 |
|  | Worst | 4 | 1.22 | 0.67, 2.23 | 0.51 | 0.53 |
|  | Worst | 2 | 1.19 | 0.55, 2.56 | 0.65 | 0.73 |
|  | Missing-successes | 3 | 0.37 | 0.08, 1.66 | 0.20 | 0.14 |
|  | Missing-successes | 2 | 0.63 | 0.24, 1.69 | 0.36 | 0.88 |
|  | Missing-failures | 4 | 0.61 | 0.36, 1.03 | 0.06 | 0.57 |
| 4-Aminoquinolines | Original | 3 | 0.05 | 0.02, 0.15 | **<0.001** | 0.29 |
| (4-As) | Original | 2 | 0.07 | 0.03, 0.16 | **<0.001** | 0.95 |
|  | Best | 3 | 0.03 | 0.01, 0.07 | **<0.001** | 0.41 |
|  | Best | 2 | 0.04 | 0.02, 0.09 | **<0.001** | 0.99 |
|  | Worst | 3 | 0.21 | 0.05, 0.91 | **0.04** | **0.007** |
|  | Worst | 2 | 0.41 | 0.15, 1.11 | 0.08 | 0.09 |
|  | Missing-successes | 3 | 0.06 | 0.02, 0.19 | **<0.001** | 0.29 |
|  | Missing-successes | 2 | 0.09 | 0.04, 0.21 | **<0.001** | 0.83 |
|  | Missing-failures | 3 | 0.11 | 0.02, 0.46 | **0.003** | **0.006** |
|  | Missing-failures | 2 | 0.19 | 0.06, 0.66 | **0.009** | **0.03** |
| Sulfanomide/ | Original | 2 | 0.20 | 0.08, 0.53 | **0.001** | 0.75 |
| Pyrimethamine | Best | 3 | 0.07 | 0.03, 0.17 | **<0.001** | 0.53 |
| (SP) | Worst | 2 | 1.98 | 1.10, 3.56 | **0.02** | 0.99 |
|  | Missing-successes | 2 | 0.21 | 0.08, 0.56 | **0.002** | 0.79 |
|  | Missing-failures | 3 | 0.56 | 0.34, 0.92 | **0.02** | 0.77 |
|  | Missing-failures | 2 | 0.57 | 0.34, 0.94 | **0.03** | 0.57 |

*Studies with 100% treatment success in both arms were excluded from analysis of original data sets and in the subsequent sensitivity analysis studies with 100% treatment success in only one arm were also excluded

**Combined ACT arms from a 3 arm study comparing 2 ACTs versus AP

***Using only the Artesunate-Mefloquine ACT arm from the study above

****Using only the Artemether-Lumefantrine ACT arm from the study above

Bold p-values are significant

**Table S3.** Summary data collected from case-report studies

| **Continuous Variables*** | **Mean** | **St Dev** | **Median** | **IQR** | **Range** | **Complete data (%)** |
| --- | --- | --- | --- | --- | --- | --- |
| Pre-parasitaemia (linear scale) | 1.9 | 2.8 | 1 | (0.3, 2.5) | (0.002, 13) | (25/36) |
| Pre-parasitaemia (log scale) | -0.44 | 2.01 | 0 | (-1.2, 0.91) | (-6.2, 2.6) | (25/36) |
| Post-parasitaemia (linear scale) | 1.8 | 1.53 | 1.3 | (0.47, 3) | (0.001, 5) | 27/36 |
| Post-parasitaemia (log scale) | -0.22 | 1.92 | 0.26 | (-0.76, 1.1) | (-6.9, 1.6) | 27/36 |
| Minimum days until recrudescence | 22.5 | 16.9 | 22.5 | (15, 28) | (2, 98) | (36/36) |
| **Categorical Variables** | **Category** | **% of all** |  |  |  | **Complete data (%)** |
| Codon 268 pre | Y (wt) | (18/36) |  |  |  | (19/36) |
|  | S/C/N (mut) | (1/36) |  |  |  |  |
| Codon 268 post | Y (wt) | (10/36) |  |  |  | (28/36) |
|  | S/C/N (mut) | (18/36) |  |  |  |  |
| Species of Malaria | *falciparum* |  |  |  |  | (17/36) |
|  | *malariae* |  |  |  |  | (1/36) |
|  | *ovale* |  |  |  |  | (1/36) |
|  | *vivax* |  |  |  |  | (1/36) |

*Pre and Post denote pre- and post- treatment parasitaemia whilst wt and mut denote wild-type Y268 and mutation 268S/C/N, respectively

**Table S4.** Minimum days until recrudescence regression analysis estimates - an interaction model on pre-parasitaemia and mutation group

| **Variable*** | **Coefficient** | **P-value** | **95%CI - Low** | **95%CI - High** | **Number Observations** | **Model adequacy** |
| --- | --- | --- | --- | --- | --- | --- |
| **Pre-parasitaemia (log)** | -0.66 | 0.68 | -4.00 | 2.68 | COMPLETE DATA (21) | 0.02 |
| **Codon 268 post (wt vs. mut)** | -17.9 | <0.001 | -26.0 | -9.83 |  |  |
| **Pre parasit. (log) x Codon 268 post** | 0.96 | 0.62 | -3.09 | 5.02 |  |  |
| **Constant** | 27.1 | <0.001 | 22.4 | 31.7 |  |  |
|  |  |  |  |  |  |  |
| **Pre-parasitaemia (log)** | -0.33 | 0.94 | -8.55 | 7.89 | OBSERVED DATA (MI) | NA |
| **Codon 268 post (wt vs. mut)** | -18.8 | 0.002 | -30.4 | -7.26 |  |  |
| **Pre-parasit. (log) x Codon 268 post** | 0.73 | 0.87 | -8.02 | 9.48 |  |  |
| **Constant** | 28.7 | <0.001 | 22.2 | 35.2 |  |  |

*Pre-parasitaemia denotes pre-treatment parasitaemia whilst wt and mut denote wild-type Y268 and mutation 268S/C/N, respectively

**Table S5.** Post-treatment parasitaemia regression analysis - an interaction model on pre-parasitaemia and mutation group

| **Variable*** | **Coefficient** | **P-value** | **95%CI - Low** | **95%CI - High** | **Number Observations** | **Model adequacy** |
| --- | --- | --- | --- | --- | --- | --- |
| **Pre-parasitaemia (log)** | 0.96 | 0.004 | 0.35 | 1.57 | COMPLETE DATA (19) | 0.57 |
| **Codon 268 post (wt vs. mut)** | -0.50 | 0.48 | -1.98 | 0.98 |  |  |
| **Pre-parasit. (log) x Codon 268 post** | -0.77 | 0.04 | -1.50 | -0.04 |  |  |
| **Constant** | 2.09 | <0.001 | 1.18 | 2.99 |  |  |
|  |  |  |  |  |  |  |
| **Pre-parasitaemia (log)** | 0.60 | 0.03 | 0.07 | 1.13 | OBSERVED DATA (MI) | NA |
| **Codon 268 post (wt vs. mut)** | -0.34 | 0.61 | -1.66 | 0.98 |  |  |
| **Pre-parasit. (log) x Codon 268 post** | -0.40 | 0.22 | -1.06 | 0.25 |  |  |
| **Constant** | 1.97 | <0.001 | 1.18 | 2.76 |  |  |

*Pre-parasitaemia denotes pre-treatment parasitaemia whilst wt and mut denote wild-type Y268 and mutation 268S/C/N, respectively

**Table S6.** Sensitivity analysis for minimum days until recrudescence regression analysis, incorporating pre-treatment parasitaemia values given as within an interval (<0.01 and <5 in Table 4 of the main text)

| **Variable*** | **Coefficient** | **P-value** | **95%CI - Low** | **95%CI - High** | **Number Observations** | **Model adequacy** |
| --- | --- | --- | --- | --- | --- | --- |
| **Pre-parasitaemia (log)** | -0.66 | 0.68 | -4.00 | 2.68 | COMPLETE DATA (21) | 0.02 |
| **Codon 268 post (wt vs. mut)** | -17.9 | <0.001 | -26.0 | -9.83 |  |  |
| **Pre-parasit. (log) x Codon 268 post** | 0.96 | 0.62 | -3.09 | 5.02 |  |  |
| **Scenario 1** |  |  |  |  |  |  |
| **Pre-parasitaemia (log)** | -0.29 | 0.80 | -2.59 | 2.02 | COMPLETE DATA (23) | 0.03 |
| **Codon 268 post (wt vs. mut)** | -18.0 | <0.001 | -25.5 | -10.5 |  |  |
| **Pre-parasit. (log) x Codon 268 post** | 0.59 | 0.70 | -2.59 | 3.77 |  |  |
| **Scenario 2** |  |  |  |  |  |  |
| **Pre-parasitaemia (log)** | -0.26 | 0.84 | -2.91 | 2.40 | COMPLETE DATA (23) | 0.03 |
| **Codon 268 post (wt vs. mut)** | -18.0 | <0.001 | -25.5 | -10.6 |  |  |
| **Pre-parasit. (log) x Codon 268 post** | 0.56 | 0.74 | -2.88 | 4.00 |  |  |
| **Scenario 3** |  |  |  |  |  |  |
| **Pre-parasitaemia (log)** | -0.22 | 0.87 | -2.95 | 2.52 | COMPLETE DATA (23) | 0.03 |
| **Codon 268 post (wt vs. mut)** | -18.1 | <0.001 | -25.5 | -10.6 |  |  |
| **Pre-parasit. (log) x Codon 268 post** | 0.52 | 0.76 | -2.98 | 4.02 |  |  |

*Complete data analyses are shown for three scenarios in which the interval values given for pre-paraseteamia are “1”, the upper values of the interval, “2”, half the upper value and “3”, a tenth of the upper value. Pre and Post denote pre- and post- treatment. wt and mut denote wild-type Y268 and mutation 268C/N/S

**Table S7.** Sensitivity analysis for post-treatment parasitaemia regression analysis, incorporating pre-treatment parasitaemia values given as within an interval (<0.01 and <5 in Table 4 of the main text)

| **Variable*** | **Coefficient** | **P-value** | **95%CI - Low** | **95%CI - High** | **Number Observations** | **Model adequacy** |
| --- | --- | --- | --- | --- | --- | --- |
| **Pre-parasitaemia (log)** | 0.96 | 0.004 | 0.35 | 1.57 | COMPLETE DATA (19) | 0.57 |
| **Codon 268 post (wt vs. mut)** | -0.50 | 0.48 | -1.98 | 0.98 |  |  |
| **Pre-parasit. (log) x Codon 268 post** | -0.77 | 0.04 | -1.50 | -0.04 |  |  |
| **Scenario 1** |  |  |  |  |  |  |
| **Pre-parasitaemia (log)** | 0.92 | 0.005 | 0.31 | 1.52 | COMPLETE DATA (20) | 0.61 |
| **Codon 268 post (wt vs. mut)** | -0.64 | 0.37 | -2.09 | 0.82 |  |  |
| **Pre-parasit. (log) x Codon 268 post** | -0.73 | 0.05 | -1.46 | 0.0009 |  |  |
| **Scenario 2** |  |  |  |  |  |  |
| **Pre-parasitaemia (log)** | 0.96 | 0.003 | 0.39 | 1.54 | COMPLETE DATA (20) | 0.44 |
| **Codon 268 post (wt vs. mut)** | -0.50 | 0.46 | -1.90 | 0.90 |  |  |
| **Pre-parasit. (log) x Codon268 post** | -0.77 | 0.03 | -1.47 | -0.08 |  |  |
| **Scenario 3** |  |  |  |  |  |  |
| **Pre-parasitaemia (log)** | 0.92 | 0.003 | 0.36 | 1.48 | COMPLETE DATA (19) | 0.58 |
| **Codon 268 post (wt vs. mut)** | -0.45 | 0.51 | -1.85 | 0.96 |  |  |
| **Pre-parasit. (log) x Codon268 post** | -0.73 | 0.04 | -1.42 | -0.05 |  |  |

*Complete data analyses are shown for three scenarios in which the interval values given for pre-paraseteamia are “1”, the upper values of the interval, “2”, half the upper value and “3”, a tenth of the upper value. Pre and Post denote pre- and post- treatment. wt and mut denote wild-type Y268 and mutation 268C/N/S

**Table S8.** Minimum days until recrudescence regression analysis estimates - an interaction model for pre-parasitaemia and mutation group - including data from an additional paper (with 6 case reports)

| **Variable*** | **Coefficient** | **P-value** | **95%CI - Low** | **95%CI - High** | **Number Observations** | **Model adequacy** |
| --- | --- | --- | --- | --- | --- | --- |
| **Pre-parasitaemia (log)** | -0.19 | 0.88 | -2.84 | 2.45 | COMPLETE DATA (27) | 0.03 |
| **Codon 268 post (wt vs. mut)** | -17.1 | <0.001 | -24.0 | -10.1 |  |  |
| **Pre parasit. (log) x Codon 268 post** | 0.50 | 0.76 | -2.87 | 3.87 |  |  |
| **Constant** | 26.2 | <0.001 | 22.7 | 29.7 |  |  |
|  |  |  |  |  |  |  |
| **Pre-parasitaemia (log)** | 0.19 | 0.96 | -6.63 | 7.01 | OBSERVED DATA (MI) | NA |
| **Codon 268 post (wt vs. mut)** | -18.2 | 0.002 | -29.2 | -7.37 |  |  |
| **Pre-parasit. (log) x Codon 268 post** | 0.18 | 0.96 | -7.22 | 7.57 |  |  |
| **Constant** | 27.8 | <0.001 | 22.3 | 33.4 |  |  |

*Pre-parasitaemia denotes pre-treatment parasitaemia whilst wt and mut denote wild-type Y268 and mutation 268S/C/N, respectively
